# ‘Failure to fail’: the teacher's dilemma revisited

**DOI:** 10.1111/medu.13772

**Published:** 2018-12-12

**Authors:** Marianne Mak‐van der Vossen

**Affiliations:** ^1^ Amsterdam University Centers VUmc School of Medical Sciences Research in Education Amsterdam the Netherlands

## Abstract

Noting that failure is a part of learning, Mak‐van der Vossen draws metaphors from the world of medical error to suggest that ‘blame‐free’ approaches to educational support must be established.

In a cross‐cutting edge article in this issue of *Medical Education*, Scarff et al.^1^ explore the literature on the ‘MUM effect’ and its relevance to performance assessment in medical education. The term ‘MUM effect’ is used to indicate that people generally prefer to keep Mum about Unpleasant Messages. Giving a fail to a medical student is such an unpleasant message. Understanding the ‘MUM effect’ could help medical educators to improve assessment in health professions education. This commentary aims to expand upon the authors’ findings by further exploring and discussing this relevant issue.

People generally prefer to keep Mum about Unpleasant Messages (MUM)

Reluctance to give a fail mark to underperforming students is well known in medical education.^2^, ^3^ This ‘failure to fail’ phenomenon was initially characterised in nursing education as a teacher's dilemma^4^, ^5^: by giving a negative grade to a student, the educator admits to having failed to effectively teach, motivate or create a learning environment for a particular student; by unjustly giving a positive grade to a student the teacher does not ensure the quality of future patient care. More recently described reasons for reluctance to fail are a lack of conceptual clarity about expectations, concern over the subjectivity of one's judgement, fear of harming a student's reputation, lack of appropriate faculty development, and uncertainty about the remediation process and its outcomes.^6^

Both the well‐being of students and the quality of future patient care call for failing a student who underperforms

Regardless of the cause, educators’ reluctance to fail is unfortunate, because underperforming students who are not identified cannot be offered assistance that would help them improve their performance.^7^ Underperformance is often caused by underlying personal or institutional factors. Elucidating such causes could be of help in creating support for the lapsing person. Furthermore, the relation of underperformance and patient safety has been made clear.^8^ Thus, identifying underperforming students is as important to the well‐being of students as it is to the quality of patient care.

Scarff et al.^1^ indicate that educators often fail to communicate appropriate feedback or ratings to their students, even after having made a proper assessment about observed unsatisfactory work. Their review reveals reasons for educators’ reluctance to speak their mind, and also possible modifying factors for this behaviour. The findings lead the authors to propose the following solutions to overcome ‘the MUM effect’: using programmatic assessment, focusing on the benefit of the message for the learner, and making delivery of undesirable messages part of the job. Furthermore, the authors propose to install specific feedback providers who are experts in delivering undesirable messages.

It may be worth simultaneously broadening the focus to more generally consider why people decide to engage or not engage in certain tasks. Helpful in this regard is the Expectancy‐Value‐Cost (EVC) model of motivation,^9^ which describes the expectancy of being successful in a task (*Can I do it?*)*,* the perceived value of engaging in a task (*Do I want to do it?*) and the costs of engaging in a task (*Are there barriers that prevent me from doing it?*). This model appeared to effectively explain the motivation of students to respond (or not respond) to lapses in professionalism they observe in medical school.^10^ It could possibly also help to understand why educators are not motivated to deliver an unpleasant message to their students. A message is unpleasant because the effect of the message is not visible for the educator (*Expectancy*)*,* there is no personal interest for the educator (*Value*)*,* and she or he has to make the effort (*Costs*). Our research showed that expectancy, value and costs are all influenced by individual and institutional factors.^10^ The individual factors align with the factors that Scarff et al.^1^ found in their literature review. The EVC model adds an explanation for the institutional factors that play a role in ‘mumming’ and ‘failure to fail’: a message is unpleasant if expectations are not clear (e.g. no opportunities for students after failing), if values and norms are not shared (e.g. norms have been imposed), and if the costs of responding are high (e.g. much time needed to observe and evaluate performance).^10^

This broadening of focus is helpful, in part, because it aligns ‘failure to fail’ with the way another quality issue in medicine has been handled, as suggested by Wong and Ginsburg: making errors.^11^ The medical community has accepted that errors inevitably will occur, and that both individual and institutional factors play a role in error making. This has enabled effective ‘blame‐free’ handling of medical errors, to enable a situation in which all stakeholders learn from errors and ultimately prevent them from happening.^11^ Given that ‘failure to fail’ has been theorised extensively, and that many practical recommendations have been proposed,^1^, ^6^, ^10^ we should not still be waiting for the change that we would like to see in practice. Rather, the time has come to acknowledge that students’ performance lapses inevitably will occur, and that they influence both the individual's well‐being and patient safety. As a result, we need to enable a ‘blame‐free’ handling of underperformance to better learn how to deal with the issues, discuss both personal and institutional causes for it, and support each other to modify such causes.

Does this mean that we have to install specific feedback givers to communicate undesirable messages? No, that would not acknowledge that lapsing is part of learning.^12^ Assessing performance and providing feedback is an essential part of the task of a medical educator. All clinical educators must be willing and able to discuss unsatisfactory performance to make their students aware of the risk. If they do so openly, focusing on the benefit to the student, it will not only have an effect on the lapsing person but also on all other students too. They will see how their educators handle underperformance, and will ultimately adopt their role models’ actions.

Lapsing is a part of learning

All clinical educators must be willing and able to discuss unsatisfactory performance

If students see their educators handle underperformance, they will ultimately adopt their role models’ actions

Lapses in performance are a part of daily life in medicine. Once we accept that performance lapses inevitably occur to everyone working in medicine, and that communicating an ‘unpleasant’ message about such lapses benefits both the lapsing person and future patient care, the teacher's dilemma will have gone.
